# Retraction: The Clinical Outcomes of Surgical Treatment of Noncontiguous Spinal Tuberculosis: A Retrospective Study in 23 Cases

**DOI:** 10.1371/journal.pone.0253035

**Published:** 2021-06-04

**Authors:** The PLOS ONE Editors

The corresponding author notified *PLOS ONE* of errors in patient data reporting in Table 1 and Fig 3 of this article [1]. They noted that Fig 3 is mislabeled and duplicates a figure published in [2], and that errors were made in collecting, recording and reporting patient data for this study [1]. The corresponding author requested the article’s retraction on behalf of the author group.

In PLOS’ internal assessment, we noted that the studies reported in [1] and [2] are partially redundant. The two articles address highly similar research questions and report overlapping conclusions and datasets. Table 1 in each article reports the same kyphotic angle data for 11 patients (participants #1, 2, 4–12 in [1]), but for six of these participants the two articles report different age or sex data. The *PLOS ONE* article reports different follow-up timepoints for these 11 participants and also reports data for participants that were not included in [2]. Nevertheless, the *PLOS ONE* Editors concluded that the degree of redundancy between the two articles is not acceptable, particularly given that the reuse of data and/or participants across the two articles was not declared.

Furthermore, there is no participant listed in Table 1 for whom the data aligns with those reported in the Fig 2 legend. This issue, the author’s comments discussed above, and the differences in demographic data reported for participants in Table 1 of [1] versus [2] call into question the overall reliability of the study’s dataset.

In light of the above concerns, the *PLOS ONE* Editors retract this article.

All authors agreed with retraction.

Fig 3 reports material from [2], published in 2012 [Springer-Verlag] and which is not offered under a CC-BY license. Fig 3 is therefore excluded from this article’s [1] license. At the time of retraction, the article [1] was republished to note this exclusion in the Fig 3 legend and the article’s copyright statement.

## References

[1] Huang J, Zhang H, Zeng K, Gao Q (2014) The Clinical Outcomes of Surgical Treatment of Noncontiguous Spinal Tuberculosis: A Retrospective Study in 23 Cases. PLoS ONE 9(4): e93648. 10.1371/journal.pone.0093648 24699518 PMC3974783

[2] Zhang H-q, Lin M-z, Shen K-y et al. (2012) Surgical management for multilevel noncontiguous thoracic spinal tuberculosis by single-stage posterior transforaminal thoracic debridement, limited decompression, interbody fusion, and posterior instrumentation (modified TTIF). Arch Orthop Trauma Surg 132, 751–757. 10.1007/s00402-012-1473-z 22350053

